# Inter-Disciplinary Work in the Context of Integrated Care – a Theoretical and Methodological Framework

**DOI:** 10.5334/ijic.7544

**Published:** 2023-06-19

**Authors:** Niamh Lennox-Chhugani

**Affiliations:** 1 International Foundation for Integrated Care, UK

**Keywords:** integrated care, inter-disciplinary teams, knowledge creation, identity, power relations, social process, institutional ethnography

## Abstract

Inter-disciplinary team working is an essential mechanism for the delivery of integrated care. This paper summarises a narrative review of the research on the ‘work’ that teams do to develop inter-disciplinary practices, addressing the question ‘How do interdisciplinary teams ‘become’ in the context of models of integrated care?’.

The narrative review identities a gap in our understanding of the active boundary work that different disciplines working together to deliver care integration engage in when creating new interdisciplinary knowledge, creating an inter-disciplinary team identity and negotiating new social and power relations. This gap is particularly significant in relation to the role played by patients and care-givers.

This paper presents a way of examining inter-disciplinary working as a process of creating knowledge, identity and power relations both in terms of a theoretical lens, circuits of power, and a methodology, institutional ethnography.

An explicit focus on understanding power relations within inclusive inter-disciplinary teams in care integration will contribute to our understanding of the gap between theory and implementation of care integration by focusing on the ‘work’ that teams do to create new knowledge.

## Introduction

Integrated care is a policy and implementation priority in health and care systems around the world [1,2]. Integrated care is an approach that aims to overcome fragmentation and deliver person-centred care within a web of care that is coordinated and continuous. Care that is integrated across organisation and even sectoral boundaries and shaped around the needs of the person at the centre, requires people from different disciplines to work together in ways they will not have historically done [3]. The ‘work’ required to make this shift in practice is vastly underestimated in implementation and may be one possible reason why implementation of integrated care is less impactful than policy makers and practitioners hope [4]. This paper aims to summarise a narrative review of the research conducted on the ‘work’ that teams do to develop inter-disciplinary practices, suggests a theoretical lens that deepens our understanding of inter-disciplinary work and proposes a complementary methodological approach that allows us to examine the ‘work’ of becoming an inter-disciplinary team in more detail. This is an important research agenda that is worthy of study.

### A narrative literature review of inter-disciplinary ‘work’

The narrative literature review aimed to provide a descriptive overview of the field on inter-disciplinary work in the context of integrated care. The search followed a series of questions. Firstly, studies explaining the concept of inter-disciplinary teams in health and care were identified. This search was refined to focus on qualitative research using a range of methods that studied the process of practicing as inter-disciplinary teams. This was further refined to focus on research studying inter-disciplinary team identity formation in the context of professional and organisational identity. The final step was a review of the literature on power relations in the context of inter-disciplinary teams.

One of the key mechanisms in the implementation of integrated care models is the multi-disciplinary, inter-disciplinary or inter-professional team [5,6,7,8]. These terms are often used interchangeably but they are different in scope and emphasis. New models of integrated person-centred care are also prompting the introduction of new roles and adaptations to skill-mix in teams with the observation that this can have mixed results for patients and staff [9,10,11,12]. Hughes et al [13] observed that multi-disciplinary working takes considerable time and effort, much of which is under-estimated and under-examined. A recent review of lessons learnt integrating care in England over the last decade noted a hope that future developments in integrated care would shift the focus from ‘integrated care’ to ‘work required to integrate’, thus enabling organisations and teams to diagnose their problems and begin to design effective solutions to integrate care [4] in an inter-disciplinary and collaborative way.

Teams have been described by Xyrichis & Ream [14] as “a dynamic process involving two or more health professionals with complementary backgrounds and skills, sharing common health goals and exercising concerted physical and mental effort in assessing, planning, or evaluating patient care. This is accomplished through interdependent collaboration, open communication and shared decision-making. This in turn generates value-added patient, organizational and staff outcomes” [14 p 238]. Collaboration is part of the process of teamwork with the common goal a defining feature. Historically teams have formed within organisations, but increasingly are formed across organisational boundaries [5].

D’Amour et al [15] define the multi-disciplinary team as members of more than one profession working independently or in parallel on the same project, coordinating their work but not necessarily meeting, whereas inter-disciplinary teams “integrate and translate, themes and schemes shared by several professions”. They share a common goal and “is based on an integration of the knowledge and expertise of each professional, so that solutions to complex problems can be proposed in a flexible and open mined way” [15 p 120]. There is a step change in the social process and effort that is required for inter-disciplinary working over and above that of multi-disciplinary working.

Interprofessional collaboration (IPC) is another term that is commonly used in health research, particularly in the context of integrated care, and it is described as an active and ongoing partnership between professionals from diverse backgrounds with distinctive professional cultures and possibly representing different organizations or sectors working together in providing services for the benefit of healthcare users [16,17]. IPC has potentially positive effects on patient health outcomes, clinical processes, efficiency outcomes and collaborative behaviour although systematic review has identified relatively weak evidence of such impact in part because of poor study design [18,19]. This definition implies that inter-professional collaboration is a social process that does not require formal structures to be enacted. Studies have focused on conceptualising the different levels and dimensions of interprofessional collaboration [20,21,22], contextual factors that influence interprofessional collaboration [15,23,24,25], the competencies required to engage in interprofessional collaboration [7] and the effects of interprofessional collaboration on teams and on patients [19,26].

The InterPACT tool [21] uses 6 dimensions of interprofessional collaboration, including (1) team goals (2) team roles and responsibilities (3) team identity (4) team commitment (5) team interdependence and (6) integration of work practices, as an initial conceptual basis for research in IPC. They also point out that the limited number of qualitative studies reporting on the implementation or evaluation of interprofessional activity leaves a significant gap in our understanding of IPC. Khan et al [27] used the Collaborative Practice Assessment Tool (CPAT) to measure ICP in primary healthcare practices in Ontario comparing Family Health Teams [FHT] and Community Health Centres [CHC]. The 7 foundations of IPC in CPAT are very similar to InterPACT, the researchers found FHTs had significantly lower scores than CHCs which they observed were partly explained by the longer establishment of CHCs. Their conclusions emphasised Importance of communication, both formal and informal, as a cornerstone of IPC.

These studies of IPC have tended to focus on the professional members of a health team, excluding social care professionals, patients and informal care-givers from the team. In the context of integrated care, where care is person-centred, this is critical gap in our understanding. Much of the research in this area to date excludes ‘non-professionals’ from the scope of collaboration.

The term inter-disciplinary is more inclusive of the wider team in the practice of people-centred integrated care. It allows for the inclusion of non-professionals and the patient and where appropriate their carers, in the team as part of the process of assessment, care planning, care management and impact assessment. Singer et al [1] captured this in their conceptualisation of interpersonal integration which refers to collaboration or teamwork among health care professionals, nonprofessional caregivers, and patients, a much more inclusive group.

Common to all of the definitions of inter-professional or inter-disciplinary team working, is the conceptualisation of it as a social process of ‘working’, organising’ or ‘becoming’ as a collective with a shared task or goal or both. It is this social process that we have relatively little understanding of specifically in the context of integrated care [4,13]. This paper summarises a narrative review of the existing research on the social process of inter-disciplinary working and proposes a theoretical and complementary methodological framework that could be used to investigate these processes in the field to help us understand how the conditions are created for high quality integrated people-centred care.

## Theory adapting: The social process of inter-disciplinary work

The starting point for much of the theoretical and empirical research in this area is the dimensions and features of the ‘active and ongoing partnership’ that D’Amour et al [15] referred to or active boundary work referred to by Schot et al [17]. Research that has explored the dimensions of inter-disciplinary working points to a number of recurring themes including informal communications, co-location, shared processes and policies, shared knowledge creation, understanding of each other roles and responsibilities, professional hierarchies and power relations, and shared clinical decision making [7,8,16,23,25,28,29]. Repeated opportunity for effective, frequent, reciprocal informal communication emerges as the single most important and tangible observable output of interprofessional collaboration [16]. Therefore we have a relatively good understanding of what creates the conditions for inter-disciplinary working but relatively less understanding of how it happens. Hughes et al [30] positions “integrated care as a social phenomenon, rather than as an intervention to be evaluated” [30 p 114], a perspective that enables us to explore the difference between how professionals experience integration (can be all-encompassing) and how patients experience it (only partial) in the context of the power relations between these groups as members, or not, of the IDT.

Best and Williams [10] noted the lack of primary research focused on the negotiated social process of inter-disciplinary team working and more recently Comeau-Vallée & Langley [31] noted the role of professional identities and power relations in the observed boundary work teams do in the context of inter-disciplinary teamwork. This paper looks at these three dimensions of inter-disciplinary working: inter-disciplinary knowledge creation, inter-disciplinary identity creation and inter-disciplinary power relations.

### Interdisciplinary knowledge creation as a social process

D’Amour et al [15] proposed that the social process of collaboration in the context of inter-disciplinary teams is based on an integration of the knowledge and expertise of each professional with the result being the creation of new knowledge. Quinlan [32] explored this further and focused on the knowledge work of multi-disciplinary teams as they create new knowledge in the context of transferring it and the application of this knowledge in clinical decision-making. What she observed was the social organisation of power including dialogical exchanges in multi-disciplinary teams that create the space for new knowledge to be created and applied. She found examples of knowledge work being mediated by hierarchy while developing a process of shared responsibility for reviewing, implementing and updating various texts such as, patient plans, policy implementation and regulations. In this early study, Quinlan found that “that the ongoing regeneration of the team’s communicative infrastructure that supports the expression of tacit knowledge *requires considerable time and energy on the part of the individuals*. In the long term, we might speculate that the process of creating new knowledge through dialogical exchange could interfere with teams’ efficiency to deliver care.” [32 p 638].

Schot et al (2019) introduced the concept of ‘active boundary work’ that professionals engage in to build common knowledge during team meetings which provides a more detailed description of the creation of new knowledge described by Quinlan (2009), the ‘active and ongoing partnership’ described by D’Amour et al (2009) and ‘work’ as described by Lewis et al (2020). Schot et al [17] identified three conceptual categories of interprofessional active boundary work: bridging gaps, negotiating overlaps and creating spaces and highlighted the work that must be done to substantiate interprofessional collaboration constantly by professionals themselves. This is particularly pertinent in integrated care where cross-organisational and sectoral work is commonplace [5].

If we explore this in the context of Xyrichis et al’s [21] dimensions of inter-professional collaboration, we can observe the creation of new knowledge within these teams as they go through the conscious or unconscious process of negotiating shared goals, understanding each other’s roles and responsibilities, working out their shared identity, commitment and interdependence as they integrate day to day clinical tasks. All of these are mediated by the professional and other identities that individuals bring into the team and the power relations between them. Working collaboratively implies smooth working relations in the face of highly connected and interdependent tasks [20]. It is likely that we will be able to observe the dynamics of this emergent inter-disciplinary identity in the context of clinical tasks.

This working collaboratively is the boundary work which has recently been defined by Langley et al [33] as “purposeful individual and collective effort to influence the social, symbolic, material, or temporal boundaries, demarcations; and distinctions affecting groups, occupations, and organizations” [33 p 704]. This boundary work has consequences for collective identity and power relations in inter-disciplinary teams. Understanding this process better will help us answer the question: How do interdisciplinary teams ‘become’ in the context of models of integrated care?

### Interdisciplinary identity creation as a social process

Team members come into an inter-disciplinary team with at least one identity already in place. Researchers have explored the role of professional identity in interprofessional collaboration and the recursive relationship between professional identities and an emergent inter-professional identity as part of the social process of active boundary work [10,31,34]. Non-professionals are rarely included in the conceptualisation of the inter-disciplinary team identity.

Currie et al [35] describe how each individual brings different personal qualities to a work-related role shaping their individual enactment of their work identity. As individuals our personal identities interact with other collective identities [36] such as professional, organisational and institutional identities in a process known as identification. Identity and identification are crucial in the post-industrial world where “identity moorings are planted in shifting sand” [37 p 14].

A recent scoping review by Wood et al [38] reviewed the evidence related to inter-professional identity in healthcare professionals. Their findings confirmed the work of previous researchers on the components or dimensions of inter-disciplinary teamwork but little additional insight into the social process of interprofessional identity creation. They highlighted studies that suggest there is a continual construction of professional and inter-professional identity through interactions and discourse that can be both conscious and unconscious. They also highlighted the lack of a clear theoretical framework conceptualising the process of identity creation, a point reinforced by Tong et al [34] who identified an over-reliance on social identity theory [36] which confines itself to the influence of group membership on group identity.

Studies looking at the process of inter-professional identity formation have identified different stages such as (1) breaking down barriers, (2) interprofessional role learning and (3) dual identity development [39] and collaborative rituals enabled through co-location leading to a shared language to build a common identity [40]. There is a body of research on the creation of organisational identity in healthcare that we can build on to develop theory about the process of inter-disciplinary team identity including the active boundary work of Schot et al [17]. Organisational or team identification has been described by Rousseau [41] as a process or “a psychological state wherein an individual perceives himself or herself to be part of a larger whole,” [41 p 217] and can be seen as an attempt by individuals to impose certainty or ontological security in highly ambiguous situations [42,43]. The process of identity transformation is viewed as a response to conflict or ambiguity between social and organisational, in this case team, and is aimed at resolving the resulting ontological insecurity about ‘who we are as a team’ [44]. This resolution leads to higher levels of commitment and well-being among team members [45], which researchers suggest, is linked to organisational and team performance [46].

Some work has been done exploring the relationship between professional identities and the process of identification [35,47,48,49,50]. This work acknowledges that professionals have multiple and competing allegiances to patients/clients, team/practice; professional groups/associations, to name a few, and explores how this impacts on their identification with a collective.

This conceptualisation of inter-disciplinary identity creation as a recursive process between individual and professional identities brings in the dynamic of power relations, particularly in the context of health and care where the relationship between knowledge and power is probably more salient that in most other contexts.

### Interdisciplinary power relations as a social process

This social process of identity creation as negotiated and active boundary work has a direct bearing on power relations in the context of inter-disciplinary teams. Researchers have underscored the mediating role of hierarchy in multi-disciplinary primary care teams [5,15,28,32,40]. A recent case study of inter-professional practice in the context of geriatric care in the Philippines [51] found what they termed interpersonal factors within the team including hierarchies and power relations were constraints on collaboration. Hughes et al [13] noted in their review of the integrated care literature using a hermeneutic approach, that many integrated care strategies had the effect of reproducing power relations between health and care professionals with evidence of the preservation of traditional hierarchies and power relationships in multi-disciplinary teams an observation reinforced by other researchers [52,53].

Power is a concept drawn on everyday in multiple contexts. We can only perceive power through its effects. Theorisations of power have varied from those that conceptualise it as a quantifiable resource that can be held and ‘stored’, to those that conceptualise it as a property of the social system in which all actors are implicated by their consent (conscious or not) to power relations [54]. Power can be both ‘power over’ or domination and ‘power to’ or capacity [55].

Post-modern approaches see power as pervasive and a function of a web of social relations [55,56,57,58]. The pervasiveness of power relations makes them difficult to resist, they are experienced as “reality” thus another way of doing things seems inconceivable. Power and knowledge are inextricably tied as knowledge is shaped by the effects of power making “truth” an unachievable objective, successful resistance will only usher in a new set of power relations. Therefore changes in knowledge can lead to changes in power relations [48,57].

For post-modernists such as Foucault [59], Callon [60] and Clegg [61], the levels are interdependent and interact constantly. Power is represented in terms of a network of power relations within which actors inscribe, embody and embed their multiple interests in material artifacts [55]. These material artifacts are resources which provide actors with the means to achieve outcomes but power is only observable in its effects, not control of the resources. Thus power serves to structure, enable or constrain the options available to actors to act by reproducing or transforming social rules or structure. The effects of these social processes can be seen in interdisciplinary teams. Mangan et al [28] touched on this in their case study examining the inter-professional working practices between general practitioners and social workers in the context of integrated care in the West Midlands of England but it was not explicitly explored. As D’Amour et al [15] noted, the absence of the patient, care-giver and family in most conceptualisations of the inter-disciplinary team is additionally problematic when considering power relations.

## Deepening our understanding of inter-disciplinary team working as a social process

The literature exploring inter-disciplinary working in the context of integrated care is still in the early stages of development. We have at least partial answers to the questions: What is an inter-disciplinary team? What makes it successful in context? What are the effects of IDT working?

We are less clear on the answer to the question: How do interdisciplinary teams ‘become’ in the context of models of integrated care? ‘Becoming’ implies a social process and this may be limited to active boundary work or it may be something more fundamental than that. We propose that taking a theoretical lens of power relations to understand how inter-disciplinary teams come together to form a shared team identity and create new inter-disciplinary knowledge, will gives us a greater understanding of the active boundary work that is being done and how is shaped by and shapes power relations.

### A theoretical framework

**Figure 1:**
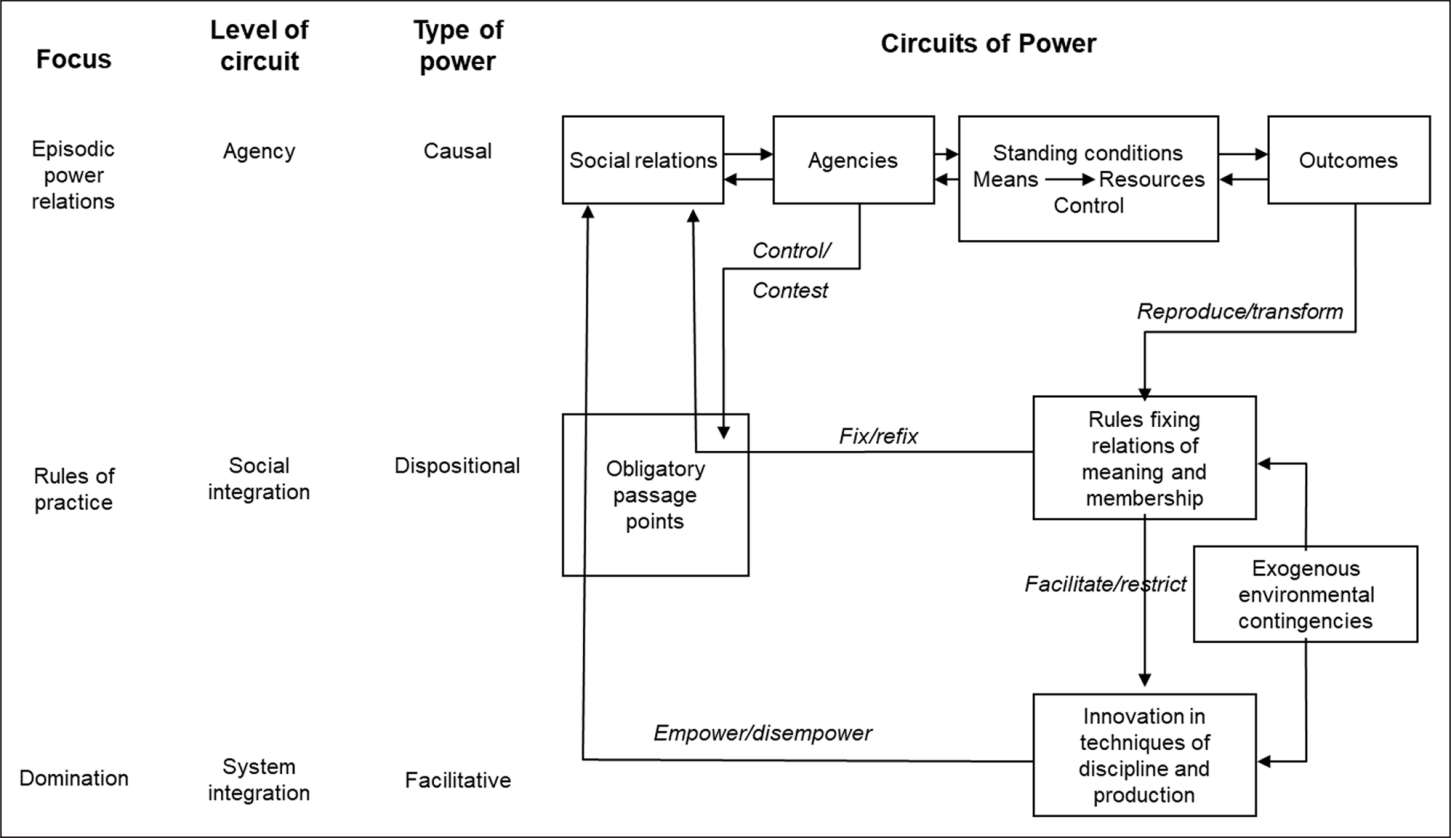
Clegg’s Circuits of Power [From Clegg, Courpasson and Phillips, (2006: 242)].

Clegg’s [61] model of power in organisations (Figure 1) to ‘represent the ways in which power may flow through different modalities’ can be used as a theoretical framework to examine how inter-disciplinary team’s social relations, rules of membership (identities) and power relations flow producing new knowledge that fixes new relations or refixes old relations. The use of the circuit’s metaphor emphasises that power is difficult to observe other than in its effects. The model enables us to “understand the different forms of agency that find expression in organizational contexts, where the players make sense of rules they actively construct and deconstruct in the context of their action” [55 p 240–241].

The level of episodic power is where the effects of power are more easily observed empirically. At this level, power is at its most visible through its enactment in social relations, agency and mobilisation of resources to achieve outcomes based on agents’ intentions. Social relations in a team are embodied in the formal and informal structures of the organisation. These structures of authority determine who is in control of resources and can mobilise them to achieve their intentions. They serve to reify power within the team and this is what most team members point to when they talk about power. We can observe this level most easily in the inter-disciplinary work of integrated care teams, in how they organise themselves using the resources made available to them relative to one another. This is level at which we start to see new forms of inter-disciplinary knowledge and identity start to emerge through the collaborative rituals identified by Lusardi et al [40] and the choices general practitioners made in whether they ‘joined’ the new inter-professional team identity. We can see this new knowledge emerge in the form of new models of care and approaches to prevention such as social prescribing, broadening the identity of the inter-disciplinary team beyond its historical health system boundaries to include public health, social care and informal care. The changing role played by patients and informal carers in new care models that explicitly create processes such as shared decision-making is observable in how resources are mobilised, for example, in the implementation of personal budgets [62].

For social and power relations to change, transformation has to happen at the second and third levels of power, dispositional and facilitative. At the level of dispositional power, the rules fixing relations of meaning and membership for a team enable agents to exercise power to achieve their ends irrespective of whether they exercise that power or not. Thus, it is a capacity, ‘power to’ rather than ‘power over’. Social rules are embedded in specific contexts and are described by Clegg et al [55] as indexical and cannot be separated from the context in which they are embedded. They constrain or limit the standing conditions within which episodic power can be exercised. In inter-disciplinary teams, this level can be observed through the meditated use of texts and formal processes. A good example of this is the multi-disciplinary team meeting where power relations are frequently exposed and sometimes negotiated [51]. The negotiated process of active boundary work that happens during these meetings in turn has the potential to change the rules of practice as a new inter-disciplinary identity emerges [32].

At the system integration level, disciplinary technologies are enacted in ways that reproduce or transform power relations that in turn empower or dis-empower agents at the level of episodic power by enhancing or limiting the range of choices available to them. Thus, discipline is both internal to the agent and external social rules or constraints, each of which mutually constitutes the other. This social system enables some social groups to dominate others and prescribe the obligatory passage points for the team where the effects of ‘power over’ can be observed. In integrated care, we see this when health and social care are ‘integrated’ but the ‘ruling’ relations of the health system still dominate. Here, the inter-disciplinary identity might be observed shaping systems of domination, in this case the rules of clinical or health practice, or potentially establishing new ones that reflect a wider field of practice, for example asset-based community development that would be more inclusive of patients and informal carers.

### A methodological approach

There are many approaches to exploring the dynamics of power relations but one of the most promising is institutional ethnography (IE). Developed by Dorothy Smith [63], a Canadian sociologist, it is a distinctive approach to research with a specific social ontology, focused on how texts and language organise people’s everyday lives with a very explicit focus on power and ‘ruling’ relations. Smith defined texts as definite forms of words, numbers or images that exist in a materially replicable form that can be reproduced across time and space and among people. The conceptual anchors of IE are (1) a starting point of observing material conditions of people’s standpoint in the everyday, what they actually do rather than think they do, (2) focusing on a broad view of ‘work’ (things that take time, effort and intent) which includes non-paid work, and (3) a presumption that how we organise socially is text based [64]. There are two levels of data collection and analysis in IE. The first involves detailed descriptions and observations of everyday work. The second, a textual analysis, involves finding the organisational details missing from the first-level experiential accounts.

IE’s focus on actions and the ruling relations that constitute and are constituted by them, make it ideal for exploring Clegg’s circuits of power in the context of inter-disciplinary team working. IE uses texts to explore how they produce and reproduce the ruling relations in institutions and observes how they influence and are influenced by everyday practice. Texts are situated in a time and place including a network of social relations, which it in turn constructs [65]. Thus texts do not just describe things but ‘do’ things. Texts reify social relations in an organisation or team by fixing individual and collective identities and constructing routines and practices [66] within identity discourses.

IE has been used in some recent studies to explicitly examine the nature of power relations in the context of person-centred coordinated care. Rowland et al [67] used this methodology to explore the experience of families in neonatal intensive care units, for example, their experience of transfers of care which surfaced fragmented communication leading to improvement initiatives including inter-professional collaboration. This methodology allowed ‘ruling’ relations to be surfaced, explored and understood in context.

Quinlan’s study [32] of knowledge work in the context of inter-professional teams in Canada used IE to explore how knowledge work was meditated by hierarchy in the context of collaborative working through texts (joint care planning). These texts were shown to both constrain inter-professional discourse but also provide the space in which professionals share tacit knowledge and create new inter-professional knowledge.

We propose that detailed descriptions and observations of everyday work alongside a textual analysis, will enable us to directly observe how inter-disciplinary teams come together to form a shared team identity and create new inter-disciplinary knowledge, give us a greater understanding of the active boundary work that is being done and fix or refix the rules that fix or refix power relations.

## Conclusion

One of the gaps in the integrated care literature is the gap between theory and implementation [11,12,68,69], that is, clear evidence that the theories of change that underpin integrated care initiatives, have the effects predicted.

Hughes [70] exposed a methodological conundrum facing implementation research in integrated care. She pointed out the challenges of using ethnographic approaches in integrated care research specifically in capturing the experiences of people at the centre of care. “Data, in the form of fieldnotes, audio recordings, transcripts and photographs, generated through participant-observation were seen as both too ‘big’ in terms of quantity and too ‘small’ in terms of generalisability…. In short, ethnography produced too much data about too few people to be of great interest to those charged with improving services and making decisions” [70 p 41]. This conundrum is likely to be at the root of our inability to close the theory – implementation gap without more and rigorous ethnographic research.

Power relations in the context of inter-disciplinary team working in integrated care are frequently acknowledged in theoretical and empirical studies but rarely explored in detail. The role played by patients and care-givers in the creation of the IDT is also absent from the literature and using institutional ethnography explicitly brings their lived experience to the fore [64]. Applying a theoretical framework of power relations and using institutional ethnography, will allow us to understand the social process of creating inter-disciplinary team identity, that is inclusive of patients, in the field of practice. The creation of integrated care knowledge and identity by inclusive inter-disciplinary teams provides a context in which this social process can be observed in practice.

As observed in the introduction to this paper, the active boundary work [4,10,17], time and energy [32] needed from teams to shift their practice to embrace inter-disciplinary working in the context of integrated care is under-researched and inadequately understood. Given its importance to the successful implementation of integrated care, this is a significant gap that needs to be addressed. What is presented here as an approach, enables us to explore this empirically in ways that can inform integrated care practice that is inclusive of patients and informal carers.
